# Acute basilar thrombosis: Recanalization following intravenous thrombolysis is dependent on thrombus length

**DOI:** 10.1371/journal.pone.0193051

**Published:** 2018-02-21

**Authors:** Hendrik Janssen, Hartmut Brückmann, Monika Killer, Suzette Heck, Grete Buchholz, Juergen Lutz

**Affiliations:** 1 Department of Neuroradiology, Ludwig-Maximilians University of Munich Hospitals, Grosshadern Campus, Munich, Germany; 2 Department for Neuroradiology, Paracelsus Medical University, Nuremberg, Germany; 3 Department of Neurolgy/Reseach Institute of Neurointervention, Paracelsus Medical University, Salzburg, Austria; 4 Department of Neurology, Ludwig-Maximilian University of Munich Hospitals, Grosshadern Campus, Munich, Germany; Fraunhofer Research Institution of Marine Biotechnology, GERMANY

## Abstract

**Introduction:**

We investigated whether thrombus length measured in Computed Tomography Angiography (CTA) is predictive of the success rate of intravenous thrombolysis (IVT) in acute basilar occlusion and whether recanalization can be achieved by additional mechanical endovascular thrombectomy.

**Methods:**

In 51 patients with acute basilar thrombosis thrombus length was measured on CTA images before intravenous thrombolysis (IVT) with rt-PA was started. After 114 minutes on average success of IVT was evaluated either by CTA or DSA. Patients with persistent basilar occlusion and no major brainstem infarction on CT underwent endovascular recanalization.

**Results:**

87% of patients had no recanalization of basilar artery after IVT alone. The average thrombus length was 15 mm in patients with persistent basilar occlusion after IVT and 7 mm in patients with recanalization after IVT. Thrombi longer than 13 mm did not resolve after IVT alone and 80% of thrombi shorter than 13 mm did not resolve either. 41 patients were transferred to endovascular recanalization; endovascular therapy was performed successfully in 90% (37 / 41).

**Conclusions:**

Recanalization rates in acute basilar occlusion after IVT alone are low and dependent on thrombus length. Additional mechanical endovascular thrombectomy showed to be a very successful recanalization therapy.

## Introduction

Acute basilar thrombosis is known to be a life-threatening disease for which fatal outcome rates of up to 90% have been reported [1, 2]. Over the last two decades intravenous thrombolysis has been established as standard treatment for acute thrombotic occlusions of intracranial vessels [3, 4]. Intraarterial thrombolysis has shown to be effective in anterior circulation MCA vessel occlusions in acute stroke [5, 6]. Brandt reported that in basilar artery (BA) thrombosis intraarterial administration of thrombolysis led to recanalization in 51%, but a mortality of 92% among the patients with persistently occluded basilar arteries was still observed [7]. The recanalization rate of basilar occlusions after IVT ranges between 53% in an analysis of different case series and 67% as reported by the BASICS Investigators [2, 8, 9]. However data on success rates of intravenously administered rt-PA for thrombolysis for acute basilar artery occlusion is so far still limited [2].

Technology and success rates of mechanical endovascular recanalization have improved over the past years [10, 11]. Recently several randomized controlled trials could prove a benefit for endovascular recanalization procedures in acute stroke [12–16]. Non-superiority of endovascular treatment was reported for acute basilar occlusions by the BASICS Investigators [8].

However previous studies have also shown that thrombus length in acute ischemic strokes of the anterior circulation is predictive of success rates of IVT [17–19]. It is still unclear whether the concept proposed by Riedel [17] is likewise valid for acute thrombosis of the basilar artery. Nevertheless thrombus length does not seem to influence success rates of mechanical thrombectomy [20, 21]. While diagnosis of basilar thrombosis can be challenging on clinical presentation alone, Computed Tomographic Angiography (CTA) has shown to be the adequate imaging modality [22, 23].

Aim of this study was to evaluate whether thrombus length as measured in CTA is predictive of success rates of intravenous thrombolysis in acute basilar thrombosis. Furthermore we investigated success of additional mechanical thrombectomy.

## Material and methods

### Study design and population

The review board of the “Bayrische Landesärztekammer” approved the retrospective study and waived requirement for informed consent.

In the retrospective analysis of our patient data from 2 university hospitals we identified 68 patients who were treated at our institutions between 2005 and 2013 and between 2016 and 2017 for acute basilar thrombosis.

We included all subjects

1. with complete occlusion of the basilar artery (BA) as proven by CT angiography (CTA) before start of intravenous thrombolysis, and
2. who received intravenous thrombolysis with rt-PA for this condition.

We excluded patients

1. with incomplete basilar occlusion,
2. who did not receive rt-PA owing to contraindications
3. with insufficient quality of CTA owing to motion artifacts or insufficient imaging protocols not allowing thrombus length measurements

### CT protocol and in-house workflow

Our diagnostic algorithm for suspected basilar thrombosis includes multislice CT with both non-enhanced computed tomography (NECT) scans of the brain and following arterial phase CTA from the aortic arch upwards with acquisition of thin slices (Slice Thickness 1 or 0.6 mm). Multiplanar reformations in sagittal and paracoronal planes were calculated.

External CT protocols of patients transferred to our institution were not standardised and included native scans in all cases and various CTA protocols. Transferred patients were re-examined by NECT and CTA on arrival at our hospitals.

For this study in-house and external CTA scans were assessed by two experienced neuroradiologists in consent regarding initial basilar thrombus length and persistent occlusion respectively. All measurements were taken with OsiriX Imaging software^®^ (Version 3.7.1 64-bit). Thrombus length was measured in sagittal or paracoronal reformations of the initial CTA before administration of IVT. To correct for tortuous vessel anatomy multiple measurements were taken in such cases parallel to the vessel walls of each segment respectively.

All externally referred patients underwent IVT before transportation and occlusion of basilar artery was reassessed on in-house CTA scans acquired after admission. In primary in-house patients occlusion of the basilar artery was reassessed on the first diagnostic DSA image series. IVT was considered successful only in complete recanalization when no residual thrombus could be seen in the basilar artery (group IVT-open). Therefore thrombus dislocation into the posterior cerebral artery (PCA) was regarded as successful IVT even if the PCA was still occluded.

Time intervals between starting the IVT and the first subsequent imaging control study as well as time between symptom onset and start of the IVT were calculated in order to rule out a bias of too short time periods for the rt-PA to take effect.

**Fig 1:**
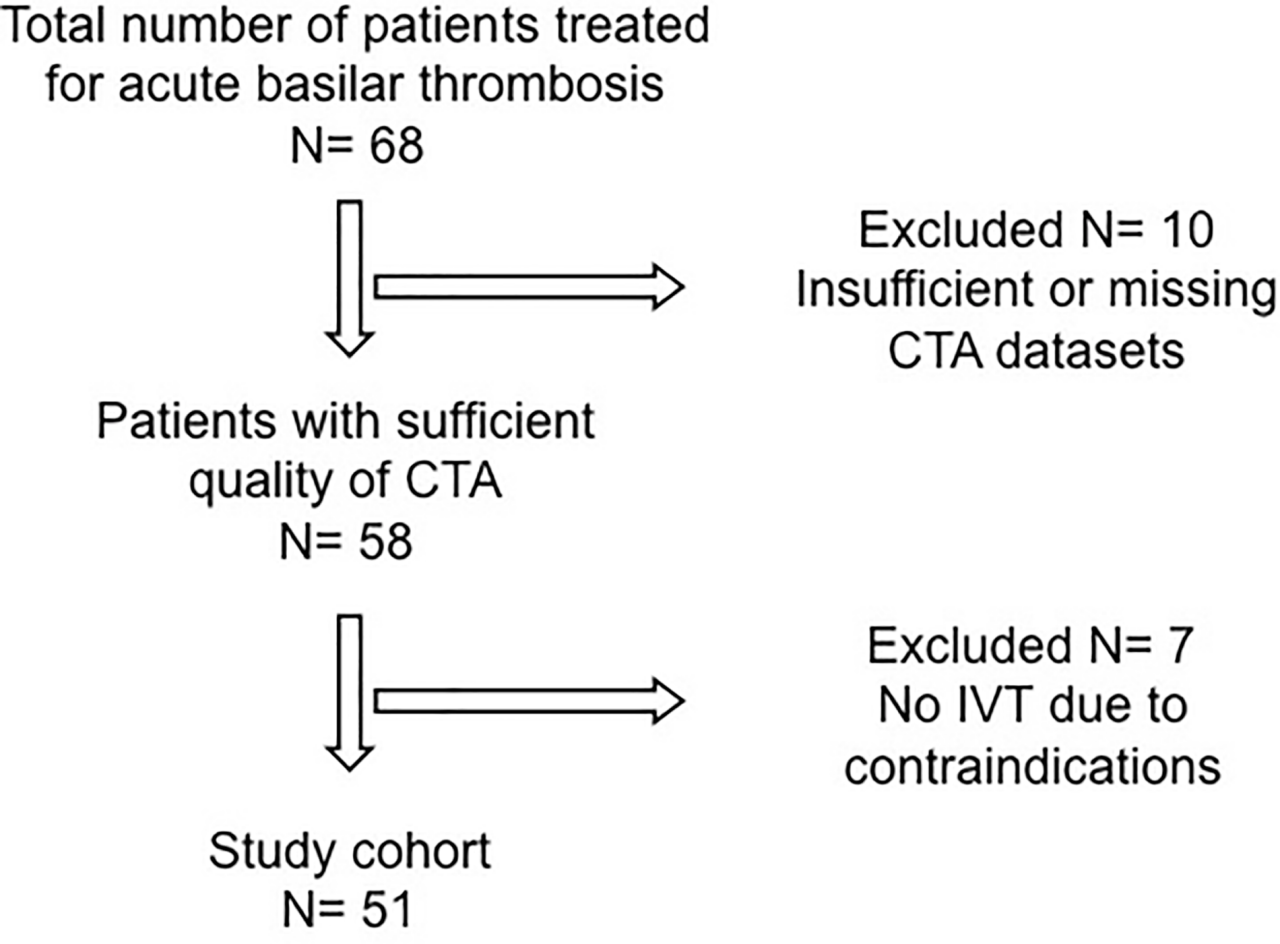
Flow chart of patient selection.

Fig 1 shows the inclusion of patients in the analysis.

### IVT and mechanical thrombectomy

As thrombolytic agent rt-PA (Actilyse^®^, Boehringer Ingelheim Pharma, Ingelheim, Germany) was given in all cases. A weight-adapted dosage was calculated with 0,9 mg / kg body weight. 10% were given as initial bolus and the rest over a 60 minutes interval. For external patients administration of lysis was started following CT before transferal and for in-house patients directly following CT.

Subsequently primary in-house patients and patients with persistent basilar thrombus were transferred to DSA for endovascular mechanical recanalization if this was reasonable considering the individual clinical condition. Mechanical recanalizations were either performed using the Penumbra Aspiration System^®^ (Penumbra Inc., Alameda, U.S.A.) or latest generation stentretriever systems such as Solitaire^®^ (eV3 Neurovascular, Plymouth, U.S.A), Preset^®^ (phenox GmbH, Bochum, Germany) or Trevo^®^ (Stryker, Kalamazoo, U.S.A.). The Penumbra Aspiration System was in use since 2005 [24] but cases before 2008 in our cohort who underwent endovascular revascularization were treated by intra-arterial thrombolysis.

### Statistical analysis

We performed all statistical analyses using SPSS Statistics 22 (IBM, Armonk/NY, USA).

Normal distribution was evaluated using the Shapiro-Wilk test. In case of normal distribution, we used a two-tailed Student’s t-test. Significance was assumed at a p value of less than 0,05. The positive likelihood ratio was calculated for recanalization of short thrombi after IVT alone.

## Results

### Study population

Among our initial cohort of 68 patients 10 patients had to be excluded due to insufficient quality of CTA that did not allow adequate thrombus length measurements. Of the remaining 58 patients another 7 did not receive IVT and were therefore excluded. The remaining 51 patients formed the final study cohort. 43 patients were included in the first and 8 patients in the second center.

Of those, 30 patients were admitted primarily to our university centers. In 21 patients diagnosis of basilar thrombosis was established clinically and by CT in surrounding district hospitals and patients were transferred to our institutions.

All patients were treated according to our in-house standardized protocols. All patients received IVT after confirmation of diagnosis by CTA. Patients with successful recanalization after IVT alone formed the group IVT-open, patients with persistent basilar occlusion after IVT alone formed the group persistent-occlusion.

### Patient characteristics

The average age of included patients was 67 y (SD 15) range 26 to 89 years. 15 (29%) patients were female. Comorbidities and clinical presentation are summarized in Table 1. The average NIHSS of all patients on admission was 21 (SD 10,5). The average mRS on discharge or transfer was 4 (SD 1,7) in all patients, median 4.

**Table 1:** Medical history, clinical presentation and outcome.

	IVT-open (n = 6)	Persistent-occlusion (n = 45)	p	All (n = 51)	excluded (n = 17)	p
**Medical history and risk factors**						
Hypertension	4 (67%)	31 (69%)	0,57	35 (69%)	11 (65%)	0,85
Diabetes mellitus	0	11 (24%)	<0,001	11 (22%)	4 (24%)	0,73
History of smoking	2 (33%)	8 (18%)	0,47	10 (20%)	4 (24%)	0,6
Hypercholesterinemia	1 (17%)	16 (36%)	0,24	17 (33%)	5 (29%)	0,92
Atrial fibrillation	1 (17%)	12 (27%)	0,53	13 (25%)	5 (29%)	0,57
**Clinical**						
NIHSS on admission	18	22	0,17	21	28	0,03
mRS on transfer or discharge	3	4	0,02	4	5	0,38
Poor outcome	4 (67%)	31 (72%)	0,39	35 (69%)	12 (71%)	0,9
Hemiparesis	3 (50%)	29 (64%)	0,45	32 (63%)	8 (47%)	0,32
Facial nerve paresis	2 (33%)	12 (27%)	0,76	14 (27%)	1 (6%)	0,07
Neglect	0	1 (2%)	0,71	1 (2%)	0	0,58
Impaired consciousness	3 (50%)	36 (80%)	0,08	39 (76%)	15 (88%)	0,16
Aphasia	0	5 (11%)	0,39	5 (10%)	0	0,19
Dysarthria	4 (67%)	24 (53%)	0,58	28 (55%)	6 (35%)	0,2
**Recanalization**						
Thrombus length (mm)	7 (SD 4)	15 (SD 9)	0,003			
Range (mm)	2–13	4–40				
IVT to imaging (min)	110 (SD 53)	115 (SD 89)	0,8			
Onset to IVT (min)	202 (SD 275)	165 (SD 167)	0,73			

Poor outcome = modified Ranking scale of 4, 5 or death; All = All included patients; excluded = Excluded patients; SD = Standard Deviation

### Time IVT and symptom onset to imaging control

The time interval between starting the IVT and the first imaging study to control success and the time interval between symptom onset and IVT are summarized in Table 1.

The vessel diameter of basilar arteries with thrombi below 13 mm that did not recanalize after IVT was 4,2 mm (SD 0,9) on average. The vessel diameter of arteries with thrombi that did recanalize after IVT was on average 3 mm (SD 0,7).

Medical history, data on clinical presentation and outcome on transfer or discharge are summarized in Table 1.

Outcome was separately calculated for all patients with recanalized basilar arteries either by IVT alone or by mechanical recanalization as opposed to patients with failed or rejected recanalization procedures. All outcome data was acquired on transfer or discharge of the patients from the treating center after 11 days on average (SD 5). Results are summarized in Table 2.

**Table 2:** Outcome in overall recanalization.

	Vessel recanalization (n = 43)	Failed vessel recanalization (n = 8)	p
**Outcome**			
NIHSS on admission	21 (SD 10)	21 (SD 11)	0,49
mRS on transferal or discharge	4 (SD 1,7)	5 (SD 1,1)	0,037
Poor outcome	28 (65%)	7 (88%)	0,28

Poor outcome = modified Ranking scale of 4, 5 or death

### Thrombus length and recanalization success

45 patients (group persistent-occlusion) (87%) had no recanalization of basilar artery after IVT alone. In 4 of those patients further treatment was not reasonable since large pontine and brainstem infarctions could be detected in CT and were confirmed by additional MRI.

The remaining 41 patients were transferred to endovascular revascularization that was performed successfully in 37 / 41 (90%), 32 with mechanical thrombectomy and 5 with IAT. In detail 35 patients underwent mechanical thrombectomy which was successful in 32 and failed in 3 (success rate 91%) In 6 patients intra-arterial thrombolysis was performed (failed: 1).

Average thrombus length in patients of the group persistent-occlusion was 15 mm (SD 9) with 4 mm being the shortest and 40 mm being the longest thrombus.

**Fig 2:**
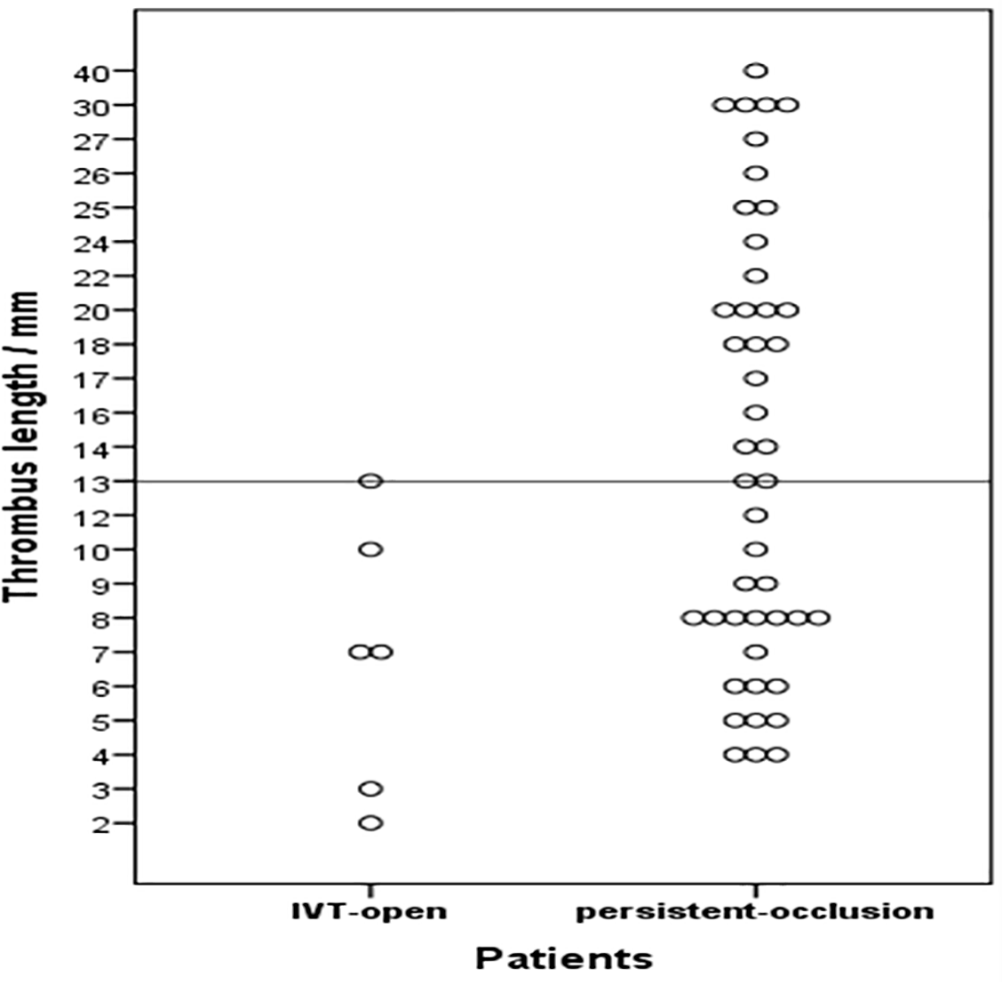
Scatterplot of all thrombus lengths in the groups IVT-open and persistent-occlusion.

24 of 45 patients (53%) of the group persistent-occlusion had a thrombus length of 13 mm or less. (Fig 2)

6 out of 51 patients (group IVT-open) (12%) showed recanalization of the basilar artery after IVT alone. Average thrombus length in this group was 7 mm (SD 4) with 2 mm being the shortest and 13 mm being the longest. (Fig 2)

## Discussion

Although IVT alone can achieve complete recanalization of basilar artery thrombosis, we found a dependency on thrombus size. A previous study showed that dissolution of thrombus by IVT alone cannot be expected in thrombus lengths of 8 mm or more in MCA occlusion [17]. In our results no thrombus longer than 13 mm resolved under IVT alone. Furthermore our data shows that short thrombi of 13 mm or less in the basilar artery have a low propensity to dissolve after IVT. In 53% of all cases with persistent thombi after IVT alone the thrombus length was 13mm or shorter. Similar to recent results for the anterior circulation [19], we cannot define a clear cut-off value in thrombus length. Even short thrombi in the basilar artery do not reliably resolve under IVT.

**Fig 3:**
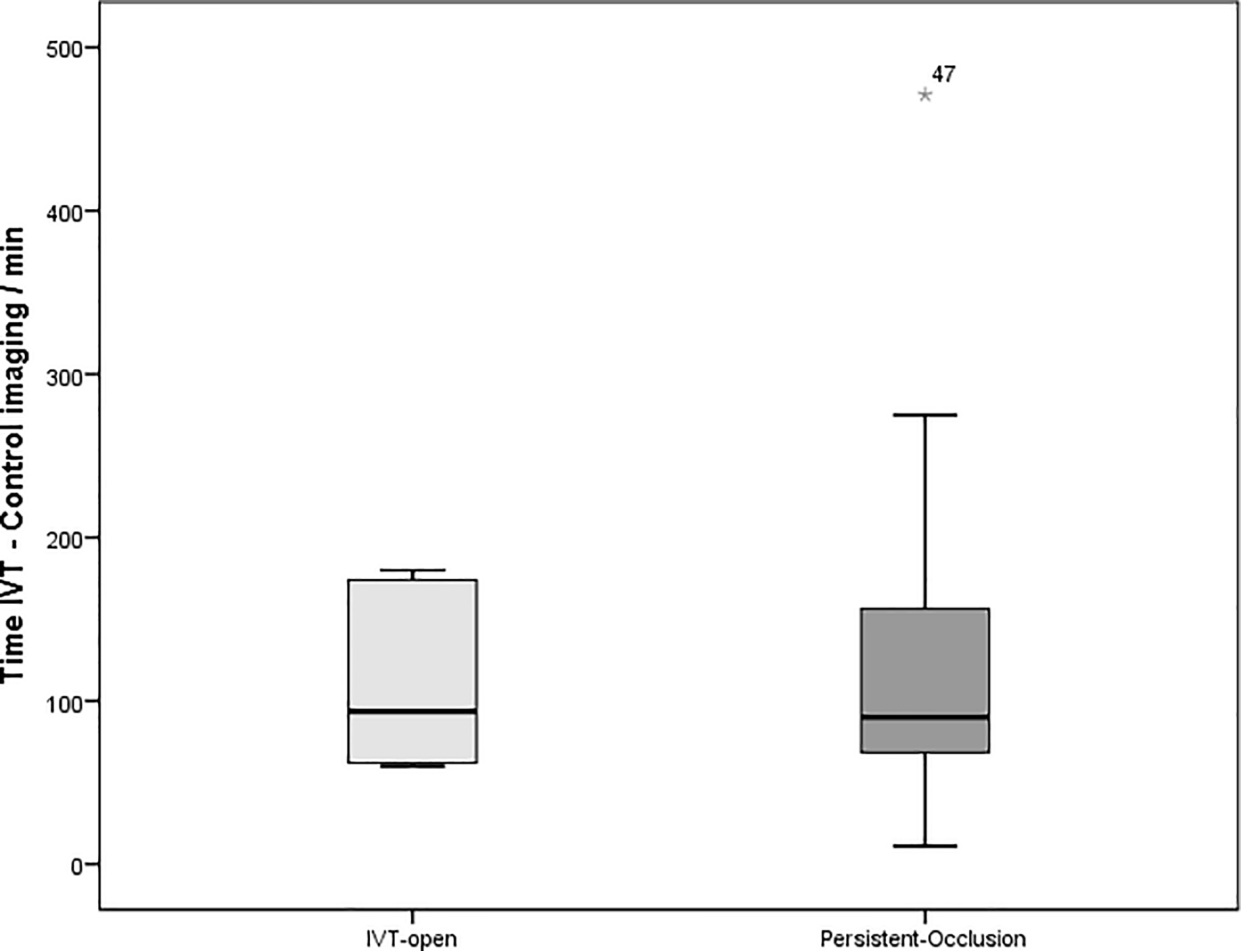
Time frames between start of i.v. thrombolysis and first imaging control for groups IVT-open and persistent-occlusion (i.v.: intravenous; min: minutes); Box: bottom = 25^th^ percentile, centerline = median, top = 75^th^ percentile, Whiskers: 1,5 x interquartile range, Star: outlier.

We could rule out a bias based on different exposure times to rt-PA between patients with successful IVT and patients with persistent occlusion (Fig 3). Patients with successful IVT had on average rather shorter rt-PA exposure times than those with persistent thrombi. Time between symptom onset and start of IVT was also not significantly different between the groups persistent-occlusion and IVT-open.

In our analysis recanalization rates following IVT alone in basilar thrombosis are low compared to the published literature [2, 8, 25–27]. However in these studies control intervals to follow up imaging are either very long or not communicated. Our study differs in this important aspect, since we have controlled the success rate of the IVT after 114 minutes on average.

We believe that the short time of rt-PA exposure until control imaging in our study is the major reason for the low recanalization rate after IVT in our data. However it is known that high rates of spontaneous recanalization occur after acute arterial occlusion within the first 24 hours [28, 29]. The half-life of rt-PA is only 4 to 5 minutes. 20 minutes after administration there is already less than 10% of the initial thrombolytic activity left. Therefore imaging control on the day after rt-PA administration as in some previous studies [19, 25, 27] would probably detect a combination of effects of IVT and simultaneously occurring intrinsic recanalization. In some studies the time to control imaging is not communicated at all [2, 8]. With an average imaging control interval around 114 min in our study, we can show a more precise course of the recanalization process and of thrombus behaviour after administration of IVT.

Looking at our data there is a considerable discrepancy between them and the results of Strbian [27] who found a recanalization rate after IVT of 62,1% in basilar thrombosis. There were two major differences in the study design. First Strbian considered partially recanalized BA as successful recanalization after IVT and we did not, as long as residual thrombus could be seen in the BA. Since many small perforating arteries of the BA supply crucial areas of the brainstem, in our opinion persistent occlusion of such perforators after partial recanalization cannot be considered as successful therapy in terms of treatment goals. Second, as mentioned before, control angiographies were performed about 24 hours after IVT in Strbian’s study. Therefore the reported recanalization rates may be biased owing to simultaneously occurring intrinsic recanalization [28, 29].

When looking at thrombus size, not only length but also the vessel diameter has to be considered. Since the surface area is proportional to the radius squared, even small differences in vessel diameter have a considerable effect on the thrombus volume. We measured vessel diameters of the BA and found them to be 29% smaller in the group IVT-open than in patients with thrombus length of 13 mm and below that did not recanalize after IVT. The basilar artery of the latter subgroup had an average diameter of 4,2 mm. This correlates with the average basilar artery size as reported in the literature [30]. The diameter of the middle cerebral artery (MCA) however is smaller on average (3 mm to 3,4 mm) [31, 32]. The surface area calculated for the vessel volume and therefore for thrombus volume would be 35% to 49% smaller in average MCA thrombi compared to our patient group persistent-occlusion with thrombi of 13 mm and below (Fig 2). The reason why IVT alone results in worse recanalization rates of the basilar artery compared to the anterior circulation remains unclear, but the different vessel size could explain the difference between our findings and anterior circulation occlusions as reported by Riedel [17]. Other data also showed that thrombus volume has an important effect on recanalization rates in intra-arterial thrombolysis. Locally applied rt-PA is less likely to resolve large thrombi entirely [33].

Since IVT is not a sufficient treatment in many basilar thrombi, the combination with subsequent endovascular mechanical thrombectomy seems to be necessary in most cases to achieve full vessel recanalization. In our series endovascular therapy had a success rate of 90% in all cases and 91% in all cases of mechanical thrombectomy. This is a high recanalization rate compared to recently reported results for acute basilar thrombosis [21]. However overall clinical outcome of the patients is still poor with mRS of 4 and worse in 71% of all patients. This is comparable to the results of the BASICS registry [8]. It has to be taken into consideration that our outcome data was acquired very early after the treatment. Late improvement was missed in our cohort due to the study design. Still successful reopening of the BA either by IVT alone or by mechanical recanalization reduced the mRS score significantly. Also the chance of poor outcome in our cohort was reduced from 88% in occluded vessels to 65% in reopened vessels. This difference was not statistically significant but this might be due to the fact that the group of permanently occluded vessels was very small.

In our data IVT alone was less successful than in the BASICS registry [8]. However time frames for control imaging after IVT were not standardized in BASICS and only 88% of the patients had imaging control at all. This creates a bias by mixing different effects leading to vascular recanalization. On the other hand endovascular therapy had a higher success rate in our series (90%) than in the BASICS registry (71%). This may in part be based on the fact that there was no defined endovascular treatment strategy in BASICS. A large percentage of the BASICS patients were treated with intra-arterial lysis and not with latest generation stentretrievers. However it has been shown that there are relevant differences in the results with different thrombectomy techniques [10, 11]. Furthermore BASICS allowed time of flight MR angiography (TOF-MRA) to confirm basilar thrombosis but it is known that this technique is insensitive to slow flow and overestimates the degree of stenoses [34]. Using this imaging technique basilar thrombosis may be feigned even if in fact a symptomatic high grade stenosis is present. Finally BASICS is a registry and not a controlled trial either and the results need to be considered with caution.

Subgroup analyses of international stroke studies suggest, that there is possibly no advantage of IVT in addition to mechanical thrombectomy in anterior circulation stroke [15]. This delicate question has to be addressed in the next years with further prospective randomized trials including acute occlusions in the posterior circulation. Surprisingly mechanical recanalization for basilar thrombosis is still not considered everywhere to be the standard of practice as reported even in current literature [27].

### Limitations

Major limitation of this investigation is the retrospective design. A large number of patients had to be excluded owing to insufficient quality or missing CTA datasets. Comparison of the excluded patients with our study cohort shows that the NIHSS on admission was higher among the excluded patients. This might be a potential bias, although outcome and all other clinical data were not significantly different between excluded and included patients. Since acute basilar occlusion is a rare entity many patients of our cohort were transferals from smaller hospitals, many CT imaging datasets in our cohort did not follow a standardized protocol.

Although we see indicators that thrombus volume is a relevant factor in this matter, we could not reliably measure thrombus volumes for this analysis. Further studies are necessary to focus on this aspect.

### Conclusion

In conclusion our study shows that in acute basilar thrombosis IVT alone is not a sufficient therapy for recanalization in the majority of cases. Additional endovascular thrombectomy is necessary to achieve this goal. Nevertheless poor clinical outcome remains a major problem, even after successful revascularization.

## References

[1] Hacke W, Zeumer H, Ferbert A, Bruckmann H, del Zoppo GJ. Intra-arterial thrombolytic therapy improves outcome in patients with acute vertebrobasilar occlusive disease. Stroke. 1988;19(10):1216–22. . 3176080 10.1161/01.str.19.10.1216

[2] Lindsberg PJ, Mattle HP. Therapy of basilar artery occlusion: a systematic analysis comparing intra-arterial and intravenous thrombolysis. Stroke. 2006;37(3):922–8. doi: 10.1161/01.STR.0000202582.29510.6b . 16439705 10.1161/01.STR.0000202582.29510.6b

[3] Hacke W, Donnan G, Fieschi C, Kaste M, von Kummer R, Broderick JP, et al Association of outcome with early stroke treatment: pooled analysis of ATLANTIS, ECASS, and NINDS rt-PA stroke trials. Lancet. 2004;363(9411):768–74. Epub 2004/03/16. doi: 10.1016/S0140-6736(04)15692-4 . 15016487 10.1016/S0140-6736(04)15692-4

[4] Hacke W, Kaste M, Bluhmki E, Brozman M, Davalos A, Guidetti D, et al Thrombolysis with alteplase 3 to 4.5 hours after acute ischemic stroke. The New England journal of medicine. 2008;359(13):1317–29. Epub 2008/09/26. doi: 10.1056/NEJMoa0804656 . 18815396 10.1056/NEJMoa0804656

[5] del Zoppo GJ, Higashida RT, Furlan AJ, Pessin MS, Rowley HA, Gent M. PROACT: a phase II randomized trial of recombinant pro-urokinase by direct arterial delivery in acute middle cerebral artery stroke. PROACT Investigators. Prolyse in Acute Cerebral Thromboembolism. Stroke. 1998;29(1):4–11. Epub 1998/01/28. . 9445320 10.1161/01.str.29.1.4

[6] Furlan A, Higashida R, Wechsler L, Gent M, Rowley H, Kase C, et al Intra-arterial prourokinase for acute ischemic stroke. The PROACT II study: a randomized controlled trial. Prolyse in Acute Cerebral Thromboembolism. JAMA: the journal of the American Medical Association. 1999;282(21):2003–11. Epub 1999/12/11. . 10591382 10.1001/jama.282.21.2003

[7] Brandt T, von Kummer R, Muller-Kuppers M, Hacke W. Thrombolytic therapy of acute basilar artery occlusion. Variables affecting recanalization and outcome. Stroke. 1996;27(5):875–81. Epub 1996/05/01. . 8623107 10.1161/01.str.27.5.875

[8] Schonewille WJ, Wijman CA, Michel P, Rueckert CM, Weimar C, Mattle HP, et al Treatment and outcomes of acute basilar artery occlusion in the Basilar Artery International Cooperation Study (BASICS): a prospective registry study. Lancet neurology. 2009;8(8):724–30. doi: 10.1016/S1474-4422(09)70173-5 . 19577962 10.1016/S1474-4422(09)70173-5

[9] Sairanen T, Strbian D, Soinne L, Silvennoinen H, Salonen O, Artto V, et al Intravenous thrombolysis of basilar artery occlusion: predictors of recanalization and outcome. Stroke. 2011;42(8):2175–9. Epub 2011/07/09. doi: 10.1161/STROKEAHA.110.605584 . 21737807 10.1161/STROKEAHA.110.605584

[10] Nogueira RG, Lutsep HL, Gupta R, Jovin TG, Albers GW, Walker GA, et al Trevo versus Merci retrievers for thrombectomy revascularisation of large vessel occlusions in acute ischaemic stroke (TREVO 2): a randomised trial. Lancet. 2012;380(9849):1231–40. Epub 2012/08/31. doi: 10.1016/S0140-6736(12)61299-9 . 22932714 10.1016/S0140-6736(12)61299-9 PMC4176618

[11] Saver JL, Jahan R, Levy EI, Jovin TG, Baxter B, Nogueira RG, et al Solitaire flow restoration device versus the Merci Retriever in patients with acute ischaemic stroke (SWIFT): a randomised, parallel-group, non-inferiority trial. Lancet. 2012;380(9849):1241–9. Epub 2012/08/31. doi: 10.1016/S0140-6736(12)61384-1 . 22932715 10.1016/S0140-6736(12)61384-1

[12] Berkhemer OA, Fransen PS, Beumer D, van den Berg LA, Lingsma HF, Yoo AJ, et al A randomized trial of intraarterial treatment for acute ischemic stroke. The New England journal of medicine. 2015;372(1):11–20. Epub 2014/12/18. doi: 10.1056/NEJMoa1411587 . 25517348 10.1056/NEJMoa1411587

[13] Saver JL, Goyal M, Bonafe A, Diener HC, Levy EI, Pereira VM, et al Stent-retriever thrombectomy after intravenous t-PA vs. t-PA alone in stroke. The New England journal of medicine. 2015;372(24):2285–95. Epub 2015/04/18. doi: 10.1056/NEJMoa1415061 . 25882376 10.1056/NEJMoa1415061

[14] Jovin TG, Chamorro A, Cobo E, de Miquel MA, Molina CA, Rovira A, et al Thrombectomy within 8 hours after symptom onset in ischemic stroke. The New England journal of medicine. 2015;372(24):2296–306. Epub 2015/04/18. doi: 10.1056/NEJMoa1503780 . 25882510 10.1056/NEJMoa1503780

[15] Goyal M, Demchuk AM, Menon BK, Eesa M, Rempel JL, Thornton J, et al Randomized assessment of rapid endovascular treatment of ischemic stroke. The New England journal of medicine. 2015;372(11):1019–30. doi: 10.1056/NEJMoa1414905 . 25671798 10.1056/NEJMoa1414905

[16] Campbell BC, Mitchell PJ, Kleinig TJ, Dewey HM, Churilov L, Yassi N, et al Endovascular therapy for ischemic stroke with perfusion-imaging selection. The New England journal of medicine. 2015;372(11):1009–18. Epub 2015/02/12. doi: 10.1056/NEJMoa1414792 . 25671797 10.1056/NEJMoa1414792

[17] Riedel CH, Zimmermann P, Jensen-Kondering U, Stingele R, Deuschl G, Jansen O. The importance of size: successful recanalization by intravenous thrombolysis in acute anterior stroke depends on thrombus length. Stroke. 2011;42(6):1775–7. doi: 10.1161/STROKEAHA.110.609693 . 21474810 10.1161/STROKEAHA.110.609693

[18] Puetz V, Dzialowski I, Hill MD, Subramaniam S, Sylaja PN, Krol A, et al Intracranial thrombus extent predicts clinical outcome, final infarct size and hemorrhagic transformation in ischemic stroke: the clot burden score. Int J Stroke. 2008;3(4):230–6. doi: 10.1111/j.1747-4949.2008.00221.x . 18811738 10.1111/j.1747-4949.2008.00221.x

[19] Rohan V, Baxa J, Tupy R, Cerna L, Sevcik P, Friesl M, et al Length of occlusion predicts recanalization and outcome after intravenous thrombolysis in middle cerebral artery stroke. Stroke. 2014;45(7):2010–7. Epub 2014/06/12. doi: 10.1161/STROKEAHA.114.005731 . 24916912 10.1161/STROKEAHA.114.005731

[20] Seker F, Pfaff J, Wolf M, Schonenberger S, Nagel S, Herweh C, et al Impact of thrombus length on recanalization and clinical outcome following mechanical thrombectomy in acute ischemic stroke. J Neurointerv Surg. 2017;9(10):937–9. Epub 2016/09/17. doi: 10.1136/neurintsurg-2016-012591 . 27634955 10.1136/neurintsurg-2016-012591

[21] Shu L, Riedel C, Meyne J, Jansen O, Jensen-Kondering U. Successful recanalization in acute basilar artery occlusion treated with endovascular therapy is independent of thrombus length. J Neurointerv Surg. 2016;0:1–7. Epub 2016/11/01. doi: 10.1136/neurintsurg-2016-012634 . 27789788 10.1136/neurintsurg-2016-012634

[22] Ng KW, Venketasubramanian N, Yeo LL, Ahmad A, Loh PK, Seet RC, et al Usefulness of CT Angiography for Therapeutic Decision Making in Thrombolyzing Intubated Patients with Suspected Basilar Artery Thrombosis. Journal of neuroimaging: official journal of the American Society of Neuroimaging. 2012;22(4):351–4. doi: 10.1111/j.1552-6569.2011.00689.x . 22303927 10.1111/j.1552-6569.2011.00689.x

[23] Puetz V, Sylaja PN, Hill MD, Coutts SB, Dzialowski I, Becker U, et al CT angiography source images predict final infarct extent in patients with basilar artery occlusion. AJNR Am J Neuroradiol. 2009;30(10):1877–83. doi: 10.3174/ajnr.A1723 . 19643923 10.3174/ajnr.A1723 PMC7051302

[24] Grunwald IQ, Walter S, Papanagiotou P, Krick C, Hartmann K, Dautermann A, et al Revascularization in acute ischaemic stroke using the penumbra system: the first single center experience. European journal of neurology: the official journal of the European Federation of Neurological Societies. 2009;16(11):1210–6. Epub 2009/08/08. doi: 10.1111/j.1468-1331.2009.02750.x . 19659754 10.1111/j.1468-1331.2009.02750.x

[25] Lindsberg PJ, Soinne L, Tatlisumak T, Roine RO, Kallela M, Happola O, et al Long-term outcome after intravenous thrombolysis of basilar artery occlusion. JAMA: the journal of the American Medical Association. 2004;292(15):1862–6. doi: 10.1001/jama.292.15.1862 . 15494584 10.1001/jama.292.15.1862

[26] Huemer M, Niederwieser V, Ladurner G. Thrombolytic treatment for acute occlusion of the basilar artery. J Neurol Neurosurg Psychiatry. 1995;58(2):227–8. ; PubMed Central PMCID: PMC1073323. 7876857 10.1136/jnnp.58.2.227 PMC1073323

[27] Strbian D, Sairanen T, Silvennoinen H, Salonen O, Lindsberg PJ. Intravenous Thrombolysis of Basilar Artery Occlusion: Thrombus Length Versus Recanalization Success. Stroke. 2014 Epub 2014/05/02. doi: 10.1161/strokeaha.114.004884 . 24781081 10.1161/STROKEAHA.114.004884

[28] Fieschi C, Argentino C, Lenzi GL, Sacchetti ML, Toni D, Bozzao L. Clinical and instrumental evaluation of patients with ischemic stroke within the first six hours. Journal of the neurological sciences. 1989;91(3):311–21. . 2671268 10.1016/0022-510x(89)90060-9

[29] Marchal G, Young AR, Baron JC. Early postischemic hyperperfusion: pathophysiologic insights from positron emission tomography. J Cereb Blood Flow Metab. 1999;19(5):467–82. doi: 10.1097/00004647-199905000-00001 . 10326714 10.1097/00004647-199905000-00001

[30] Saeki N, Rhoton AL Jr. Microsurgical anatomy of the upper basilar artery and the posterior circle of Willis. J Neurosurg. 1977;46(5):563–78. Epub 1977/05/01. doi: 10.3171/jns.1977.46.5.0563 . 845644 10.3171/jns.1977.46.5.0563

[31] Umansky F, Juarez SM, Dujovny M, Ausman JI, Diaz FG, Gomes F, et al Microsurgical anatomy of the proximal segments of the middle cerebral artery. J Neurosurg. 1984;61(3):458–67. Epub 1984/09/01. doi: 10.3171/jns.1984.61.3.0458 . 6747682 10.3171/jns.1984.61.3.0458

[32] Vuillier F, Medeiros E, Moulin T, Cattin F, Bonneville JF, Tatu L. Main anatomical features of the M1 segment of the middle cerebral artery: a 3D time-of-flight magnetic resonance angiography at 3 T study. Surgical and radiologic anatomy: SRA. 2008;30(6):509–14. doi: 10.1007/s00276-008-0360-3 . 18465079 10.1007/s00276-008-0360-3

[33] Schulte-Altedorneburg G, Hamann GF, Mull M, Kuhne D, Liebetrau M, Weber W, et al Outcome of acute vertebrobasilar occlusions treated with intra-arterial fibrinolysis in 180 patients. AJNR Am J Neuroradiol. 2006;27(10):2042–7. Epub 2006/11/18. . 17110663 PMC7977195

[34] Ozsarlak O, Van Goethem JW, Maes M, Parizel PM. MR angiography of the intracranial vessels: technical aspects and clinical applications. Neuroradiology. 2004;46(12):955–72. Epub 2004/12/08. doi: 10.1007/s00234-004-1297-9 . 15580489 10.1007/s00234-004-1297-9

